# Pharmacists’ roles and perceptions in managing prenatal exposure to medications: A qualitative study

**DOI:** 10.1097/MD.0000000000045618

**Published:** 2025-10-31

**Authors:** Fahad S. Alshehri, Nasser M. Alorfi, Ahmed M. Ashour, Saad M. Wali, Moosa H. Atwadi, Abdulaziz Mohammad Alghamdi, Mohammed M. Aldurdunji, Shaker T. Alsharif

**Affiliations:** a Pharmacology and Toxicology Department, College of Pharmacy, Umm Al-Qura University, Makkah, Saudi Arabia; b King Salman Center for Disability Research, Riyadh, Saudi Arabia; c Pharmaceutical Care Administration, Jeddah First Health Cluster, Ministry of Health, Jeddah, Saudi Arabia; d Althager General Hospital, Ministry of Health, Jeddah, Saudi Arabia; e East Jeddah Hospital, Ministry of Health, Jeddah, Saudi Arabia; f Pharmaceutical Practices Department, College of Pharmacy, Umm Al-Qura University, Makkah, Saudi Arabia; g Pharmaceutical Sciences Department, College of Pharmacy, Umm Al-Qura University, Makkah, Saudi Arabia.

**Keywords:** maternal health, medication counselling, pharmacists, pregnancy, prenatal medication safety

## Abstract

The safe use of medications during pregnancy is a critical aspect of maternal and fetal health. Pharmacists play a key role in medication management by providing counseling, assessing risks and benefits, and supporting healthcare providers. However, knowledge gaps, lack of consistent guidelines, and patient hesitancy may reduce their ability to offer effective guidance. This study examines pharmacists’ perceptions, roles, and challenges in managing prenatal medication exposure. A qualitative research approach was employed using a self-administered online survey. The study targeted pharmacists working in hospitals, mental health facilities, and community pharmacies who had experience counseling pregnant women about medication safety. Thematic analysis was conducted following Braun and Clarke framework to identify key themes emerging from participants’ responses. Eighteen pharmacists participated in the study, with experience ranging from 1 to 24 years. Three major themes emerged: training and knowledge gaps, where pharmacists reported limited formal training on prenatal medication management and a reliance on self-education and online resources; pharmacists’ roles and responsibilities, highlighting their role in risk assessment, patient counseling, and collaboration with healthcare providers, though their contributions were often underutilized; and challenges and barriers, which included patient fears, misinformation, and the lack of standardized guidelines. Additionally, pharmacists emphasized the need for training programs, access to up-to-date drug safety resources, and interdisciplinary collaboration to enhance their competency in advising pregnant women. Pharmacists play a vital role in ensuring medication safety during pregnancy but face challenges due to training deficiencies, patient hesitancy, and lack of clinical guidelines. Addressing these gaps through enhanced training, evidence-based resources, and interdisciplinary collaboration can improve maternal healthcare. Future efforts should focus on targeted educational programs to strengthen pharmacists’ role in prenatal medication management

## 1. Introduction

The safety and efficacy of medication use during pregnancy remain high concerns in maternal and fetal healthcare.^[1]^ Pregnant women frequently require pharmacological treatment for preexisting conditions such as hypertension, diabetes, and epilepsy, as well as pregnancy-induced complications, including gestational diabetes and preeclampsia.^[2–6]^ However, the physiological changes that occur during pregnancy can alter drug pharmacokinetics and pharmacodynamics, potentially affecting both maternal health and fetal development.^[7]^ While some medications are essential during pregnancy, medications may have teratogenic risks or lead to complications such as preterm birth, intrauterine growth restriction, or congenital anomalies.^[8,9]^ Given these difficulties, ensuring the rational and safe use of medications during pregnancy is highly important.

Pharmacists play an important role in advising pregnant women and healthcare providers on medication safety.^[10]^ Therefore, pharmacists provide medication counseling, assess risk–benefit profiles, and offer evidence-based recommendations to optimize treatment outcomes.^[11]^ Their expertise is particularly important in reducing the risks associated with self-medication, over the counter drug use, and misinformation obtained from non-medical sources such as the internet or social media.^[12]^ In addition, pharmacists often encounter challenges in providing effective guidance on prenatal medication use. Studies have shown that pharmacists may experience knowledge gaps regarding teratogenic risks, a lack of standardized guidelines, and limited access to updated drug safety information specifically for pregnant women.^[13,14]^ Additionally, communication barriers and patient-related concerns may further complicate pharmacists’ ability to provide comprehensive counseling.^[10]^

Even though previous studies have explored some aspects of medication safety in pregnancy, there remains a gap in understanding how pharmacists perceive their role in managing prenatal medication and the challenges they encounter in clinical practice.^[15,16]^ Most studies have largely focused on physicians’ prescribing behaviors or pregnant women’s medication use patterns, with limited attention given to pharmacists’ perspectives.^[17,18]^ Understanding pharmacists’ experiences and identifying barriers to effective counseling are essential steps in improving maternal healthcare services and optimizing medication safety during pregnancy.

This study aimed to explore the roles, perceptions, and challenges of pharmacists in managing prenatal exposure to medications. By using a qualitative research approach, this study provided in-depth understandings regarding pharmacists’ experiences, identified existing limitations in current pharmaceutical practices, and suggested strategies to enhance pharmacists’ contributions to prenatal medication management. The findings could enhance pharmacist interventions for pregnant women and support the role of pharmacists in maternal healthcare.

## 2. Methodology

### 2.1. Study design

This study performed a qualitative research design to explore pharmacists’ perceptions, roles, and challenges in managing prenatal medication exposure. A phenomenological approach was used to gain in-depth insights into pharmacists’ experiences and professional perspectives regarding medication safety during pregnancy. This design allowed for an in-depth exploration of participants’ responses and thematic patterns emerging from their experiences.

### 2.2. Study setting and participants

The study was conducted among pharmacists working in various healthcare settings, including hospitals, mental health facilities, and community pharmacies. Participants were selected through purposive sampling, ensuring representation from different pharmacy sectors and experience levels. Participants were recruited through purposive sampling by distributing the survey link through professional pharmacy networks and social media. Of the 26 pharmacists invited, 18 completed the survey, resulting in a response rate of approximately 69%.

#### 2.2.1. Inclusion and exclusion criteria

Pharmacists were eligible to participate if they were licensed and actively practicing in a healthcare setting, had experience providing medication-related counseling to pregnant women, and were willing to participate in a survey-based study. However, pharmacists who had no direct experience with prenatal medication counseling or were not currently practicing were excluded from the study.

### 2.3. Data collection

Data were collected through a self-administered online survey distributed via Google Forms, allowing participants to complete the questionnaire at their convenience. A survey link was sent to eligible pharmacists, providing them with the flexibility to respond at their own pace. The survey consisted of open-ended questions, enabling participants to share detailed insights based on their experiences. The questionnaire was designed to explore key aspects of pharmacists’ roles in prenatal medication management, including their training and knowledge of medication use during pregnancy, perceived responsibilities in advising pregnant women, methods used for risk assessment and decision-making, challenges and barriers encountered in counseling, and resources and training needs to enhance their competency in this area. To ensure thoughtful and comprehensive responses, participants were given sufficient time to complete the survey. The online survey was preferred over interviews or focus groups due to the wide geographic distribution of participants in the kingdom and varying work schedules. This format allowed participants to respond at their convenience and provide detailed answers. The 10 open-ended questions addressed training and knowledge, perceived roles, risk–benefit assessment, counseling examples, challenges, barriers, and recommendations (full questionnaire and data in File S1 and File S2, Supplemental Digital Content, https://links.lww.com/MD/Q521; https://links.lww.com/MD/Q522, respectively).

### 2.4. Data analysis

The responses were analyzed using thematic analysis.^[19]^ A qualitative design was used to explore pharmacists’ perspectives on prenatal medication management, similar to the approach.^[20]^ The analysis followed Braun and Clarke thematic framework and was conducted inductively, allowing themes to emerge naturally from the data without pre-established categories. The process began with researchers thoroughly reading all responses multiple times to gain familiarity with the content. Initial codes were then generated by identifying key phrases and concepts, which were organized using Microsoft Excel. These codes were grouped into broader categories, from which overarching themes were developed. Two researchers independently coded the data and collaboratively refined the coding framework. Although formal intercoder reliability was not calculated, consistency was assured through independent coding by 2 researchers, followed by iterative discussions to resolve discrepancies and refine the coding framework until full agreement was reached. Thematic saturation was considered achieved when no new codes or themes emerged from the final 5 responses, consistent with established standards for qualitative studies involving focused research questions and relatively homogenous samples. The thematic saturation was examined when analysis of the final 5 responses yielded no new codes or themes. This review included participants from varied practice settings (hospital, community pharmacy, and mental health facilities) and with diverse years of experience (1–24 years) to ensure thematic diversity. Responses were compared across these backgrounds to confirm that core themes were consistent across different contexts.

### 2.5. Ethical considerations

The study was approved by The Biomedical Research Ethics Committee at Umm Al-Qura University (Approval Number: HAPO-02-K-012-2025-01-2472). Informed consent was obtained from all participants before data collection, and pharmacists were informed that their participation was voluntary. Before accessing the questionnaire, participants were presented with an online consent statement outlining the study’s purpose, procedures, and confidentiality measures. They were required to select “I agree” to proceed, indicating explicit informed consent. To maintain confidentiality, all responses were anonymous, and no personally identifiable information was collected. Additionally, all data were securely stored and accessible only to the research team, ensuring participant privacy and data protection.

## 3. Results

**Figure 1.**
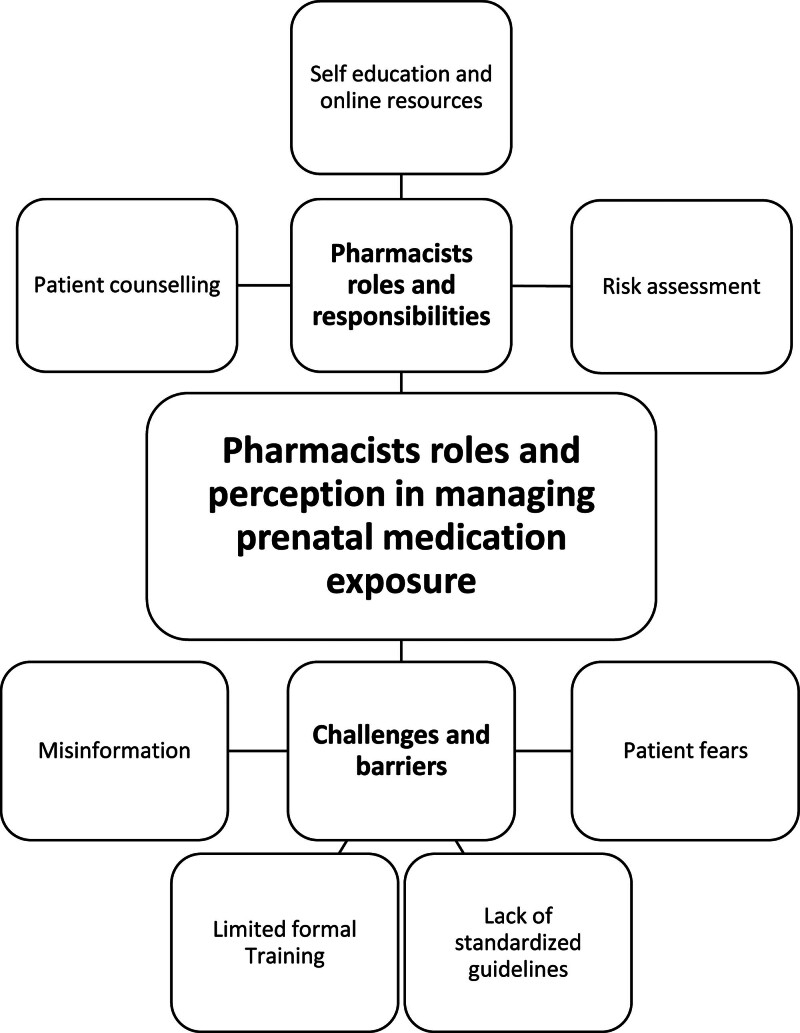
Thematic map of pharmacists’ perceptions on prenatal medication management. The map was constructed following Braun and Clarke thematic analysis framework, illustrating the relationships between the 3 major themes (training & knowledge gaps, pharmacists’ roles & responsibilities, and challenges & barriers) and their associated subthemes, as derived from participants’ responses.

The qualitative analysis identified 3 major themes related to pharmacists’ perspectives on managing prenatal medication exposure: training and knowledge gaps, pharmacists’ roles and responsibilities, and challenges/barriers in counseling pregnant women. These themes and their interconnections are illustrated in the thematic map (Fig. 1).

### 3.1. Demographic characteristics of study participants

A total of 18 pharmacists participated in this study, representing several healthcare settings, including hospitals, mental health facilities, and community pharmacies. The participants held roles such as clinical pharmacists, pharmacy supervisors, and medication safety officers, with experience ranging from 1 to 24 years, as shown in Table 1.

**Table 1:** Demographic characteristics of study participants.

Gender	Work place	Role	Years of experience
Female	Hospital	Clinical Pharmacist	10 yr
Female	Mental Health Healthcare Facility	Pharmacist	16 yr
Male	Hospital	Consultant Pharmacist	15 yr
Male	Hospital	Clinical pharmacist	10 yr
Male	Hospital	Senior Pharmacist	15 yr
Female	Hospital	Medication safety officer	4 Years
Female	Hospital	Inpatient pharmacist	10 yr
Female	Hospital	Pharmacist	5 yr
Male	Hospital	Pharmacy supervisor	15 yr
Male	Hospital	Pharmacist	10 yr
Male	Hospital	Pharmacist	24 yr
Male	Hospital	Pharmacist	23 yr
Male	Hospital	Pharmacist	11yr
Male	Community pharmacy	Community Pharmacist	3 yr
Female	Hospital	Pharmacist	1 yr
Female	Hospital	Pharmacist	2 yr
Female	Hospital	Pharmacist	3 yr
Female	Community pharmacy	Pharmacist	2 yr

The gender, workplace, professional role, and years of experience of the 18 pharmacists who participated in the qualitative study on prenatal medication management.

### 3.2. Training and knowledge gaps

Most pharmacists reported limited or no formal training on prenatal medication management. While some had exposure during their university education, there was a lack of structured post-graduate training. Many relied on self-education, personal experience, and online resources to guide their practice.

*“There is no self-training, only what I learned during my university studies.”*

*“I have been exposed to limited training, but there is a need for more structured programs on medication safety in pregnancy.”*

Pharmacists highlighted the importance of up-to-date, evidence-based training programs to enhance their competence in managing medication safety for pregnant women. The thematic analysis of pharmacists’ responses regarding prenatal medication management is summarized in Table 2.

**Table 2:** Thematic analysis of pharmacists’ perceptions on prenatal medication management.

Training received	Perceived role	Responsibilities	Risk and benefit assessment	Advising pregnant women	Challenges	Barriers	Needed resources	Recommendations
There is no self-training only.	It’s okay.	Verify information, damages and find alternatives.	The full need for medication should be considered and the pros and cons should be weighed.	I don’t remember.	Convincing them of the harm of some common medicines.	There is none.	Intensifying education in hospitals and holding courses.	Effective training.
During study and training then self-education.	An important and influential role	In which months of pregnancy – Allergy – Its effect on the fetus – Drug classification – Its conflict with food or other medications	Based on the patient’s tolerance for pain or symptoms vs the benefit.	Do not take calcium and iron at the same time – take iron on an empty stomach.	Lack of familiarity with some medications and their classification.	No.	There is none.	Intensive training especially for those working in maternity and children’s hospitals.
Training during university studies at all stages... in addition to working in the Ministry of Health hospitals, especially the Maternity and Children’s Hospital, during one of the career progressions stages... where it was important to know all the pharmaceutical information related to the safety of medicines during pregnancy. Many workshops and scientific lectures were implemented at that time, in addition to setting therapeutic and preventive policies and procedures to reduce medication errors or negative impact on pregnancy.	1. Providing pharmaceutical advice in all its details. 2. Ensuring the safety of medications and the extent of their safety in using the medication during pregnancy, in addition to calculating doses, drug interactions, etc. 3. Educating about nutritional supplements used during pregnancy. 4. Monitoring chronic diseases in pregnant women and the extent of preparing the treatment plan in light of that. 5. Awareness of side effects. 6. Coordinating with health care providers at all stages of pregnancy regarding the treatment plan for the pregnant woman.	1. Medication knowledge: Advising pregnant women on medications and appropriate dosages. 2. Prescription details: Written to ensure that the prescribed medications are free of maternal or fetal blood. 3. Nutrition education: Special guidance and appropriate dosages. 4. Chronic disease status: Assisting pregnant women in managing their chronic condition safely. 5. Side effects awareness: Alert pregnant women to certain side effects and how to manage them. 6. Compliance with regulations and legislation: Required for local laws regarding medication separation, especially those related to abortion. 7. Communication with healthcare providers: Still with indications and compatibility of medications with pregnancy.	Through studies, research and information that are published and approved in this regard, in addition to scientific committees in this regard.	Folic acid: Recommended during the first 3 months to prevent birth defects. Paracetamol: A safe pain reliever and fever reducer when needed. Iron supplements: Prescribed to treat iron deficiency anemia that may accompany pregnancy.	1. Lack of adequate information. 2. Physiological changes. 3. Effective communication. 4. Anxiety and fears. 5. Cultural and social diversity. 6. Changes in health status.	1. Hormonal changes and mood swings 2. Anxiety and psychological stress 3. Health challenges 4. Social and media influences.	1. Careful review of drug safety classification during pregnancy. 2. Use of reliable drug databases. 3. Continuous communication with the medical team. 4. Educating the pregnant woman. 5. Evaluating risks and benefits. 6. Periodically updating medical information.	1. Updating medical information. 2. Continuous communication with the medical team. 3. Educating the pregnant woman from a pharmaceutical perspective. 4. Assessing risks and benefits. 5. Providing correct and comprehensive advice. 6. Providing information about care during pregnancy from a therapeutic perspective.
I have been exposed to training that provides information about the medication which should be avoided during pregnancy	Our role as a pharmacist is to ensure the quality use of medications (appropriate, effectiveness and safety)	We should consider the pregnancy period (in which trimester) as well as risk and benefit of exposure to such medications	The diagnosis should be mentioned clearly	Honestly, I don’t have an example to be applicable for this question	The patient feel that she could harm her baby	I don’t think so	There are many reliable resources that can be used while dealing with pregnancy	They should use reliable resources and assessment tool to be able to apply their knowledge and skills towards these patients while they are dealing with
I have completed training sessions on prenatal pharmacology, including workshops on the **Pregnancy and Lactation Labelling Rule (PLLR)** and participation in courses focused on the safe use of medications during pregnancy. Additionally, I frequently use resources such as **Micromedex**, **UpToDate**, and **LactMed** for ongoing learning.	My primary role is to assess the safety of prescribed medications for both the mother and fetus. This includes evaluating teratogenic risks, ensuring compliance with evidence-based guidelines, and providing counseling to patients about the importance of adhering to prescribed treatments to manage health conditions during pregnancy.	Key responsibilities include: 1. Evaluating medication safety for both mother and fetus using reliable databases. 2. Clearly communicating risks and benefits of medications. 3. Collaborating with other healthcare providers to optimize treatment plans. 4. Monitoring for adverse effects and ensuring patient understanding of the prescribed therapy.	I use tools like **FDA Pregnancy Categories**, **PLLR**, and databases such as **Micromedex** to analyze safety data. I weigh the potential benefits of treating maternal health conditions against the risks to fetal development. Collaboration with the physician is critical for deciding on the safest and most effective treatment option.	A patient with epilepsy was on **valproic acid**, which has a high risk of teratogenic effects. I collaborated with her physician to switch her to **lamotrigine**, which is safer during pregnancy. I explained the importance of adherence to prevent seizures while minimizing risks to the fetus and monitored her progress throughout the pregnancy	Common challenges include: – Lack of sufficient clinical data for certain medications. – Patients’ reluctance to take medications due to fear of harming the fetus. – Miscommunication or conflicting advice from non-medical sources. – Balancing risks of untreated maternal conditions with potential medication side effects.	Yes, barriers include: – Limited health literacy or misunderstanding of medical information. – Cultural beliefs and stigma surrounding medication use during pregnancy. – Time constraints in counseling sessions. – Inconsistent access to evidence-based resources for both patients and providers.	1. Access to updated, evidence-based databases such as **LactMed**, **Micromedex**, and **UpToDate**. 2. Training in motivational interviewing techniques for counseling. 3. Easy-to-use reference charts or mobile applications for pregnancy-specific medication guidance. 4. Interdisciplinary case discussions to share best practices.	Improvements could include: – Specialized training programs focusing on pregnancy and lactation pharmacology. – Enhanced collaboration with obstetricians and other healthcare providers. – Development of patient education materials in multiple languages and formats. – Implementation of tele pharmacy services to extend access to counseling.
During drug information rotation in National Guard Hospital in Jeddah	Important and effective	The safety and efficacy of medication prescribed for pregnant women	checking pregnancy category of each medication prescribed, using updated and reliable references	Advise them to take calcium & ferrous separately to avoid drug interaction and have the benefit of supplements	Nothing	Complete and updated medication history	Effective communication skills, Drug awareness and education	Take responsibility for the importance of pharmacist role in this matter and follow up on providing effective and safe medications for pregnant women.
No specific training about medication management during pregnancy.	Evaluate the use of medicines in pregnancy safely and efficiently.	Ensuring that medications are safe according their pregnancy stage.	By using available evidence-based information.	Give them an ideal schedule for using their vitamins (time, before or after meal)	The effect of social media for their knowledge and level of anxiety makes the counseling more complicated.	Insufficient time to communicate information to pregnant women in out-patient pharmacy.	Specific references	Enhancing a role of DIC to provide a verbal or written information about any drug the pregnant may need.
I have not received training, practical and scientific experience.	An important role for the pharmacist in hospitals, especially women’s and maternity hospitals. The pharmacist must have knowledge and understanding of medicines. In the event of lack of knowledge, references must be available to search for and easily accessible.	Explanation of how to use the medicine and its safety for pregnant women	With knowledge and in case of lack of knowledge with research	Calcium and iron are separated. How to use them and how to separate them is explained.	Fear and disbelief of pregnant women	Not trusting the pharmacist	Drug Information Center Information Resources	Training courses for new pharmacists, continuous lectures to enhance pharmaceutical knowledge
Nothing	Essential	Teratogenicity	Depending on the medication category and availability of alternatives and severity of the symptoms	It varies from medicine to another e.g.: don’t use this medication in second trimester	Not knowing his medications	No	Source of information e.g., Micromedex, UpToDate, Lexicomp.	Easy accuse to resources
Referring the case to a doctor following up on the case and communicating with doctors in the event that medications are prescribed that have side effects on the pregnant mother or the fetus.	An effective role in preserving the health and safety of future generations and working to prevent diseases that harm the environment and society	Do not use mixtures or any treatment without consulting a doctor to avoid unwanted complications.	Prescribed medications for pregnant women are effective and harmless to the pregnant woman or the fetus.	For example, do not use calcium tablets with iron tablets due to poor absorption.	Refusal to follow instructions on how to use the medication	No, but some obstacles come from outside the health framework.	Follow up on developments and updates on medicines for pregnant women	Intensify guidance through media and social communication
There is no training	hard-working	Make sure that the medicine does not harm the fetus or the pregnant woman during all stages of pregnancy.	There are risks if you don’t pay attention.	Thyroid medications, when is the best time to take them, and also the interaction of calcium with iron?	Some pregnant women are not responding or caring.	I am worried about the pressure of work and not intensifying the courses in this regard.	Training courses and resources	The density of courses and communication between pharmacists in obtaining information from its source
Online&clinical	Most important to avoid fetus malformation	Asking which trimester, she is and about home medication	check if the medicine helps the mother more than it could harm the baby. They look at the stage of pregnancy, medicine safety, and expert advice.	For a pregnant woman with high blood pressure: I recommend labetalol or methyldopa because it’s safe during pregnancy. Treating your blood pressure protects both you and your baby from complications like preeclampsia	Other challenges: Ensuring patient understanding, addressing unplanned pregnancies while on medication, and navigating limited alternatives for certain conditions.	inconsistent information, limited availability of safe medications, and pressure for quick prescription filling	Helpful tools include drug databases (Lexicomp), pregnancy guidelines (ACOG), patient education materials, and clinical decision support systems.	better collaboration with healthcare providers pregnancy-specific training, better communication tools, updated drug databases, and more consultation time (Enough staff) female staff best to give counsel to pregnant
	An important role as a first line defence against the dangers of using medications that harm the mother or her baby.	That it will not harm her or the baby. And it is preferable for her in terms of route of administration, side effects profile, and patient psychological state.	For me I prefer not to take any medication unless strongly recommended. Because some women may take overdoses by mistake or take the wrong medication that will result in a problem. If she had a critical or chronic condition, I must give her a comprehensive background about the medications that she’s using	Once a pregnant woman came to me and asking about vitamins, when I give her a certain product, I asked her if she is taking anything else. She said she is taking another type of multivitamins and she takes iron. She doesn’t know if that is enough if she takes those supplements and follow a healthy eating habits and healthy lifestyle. If I didn’t ask her and give her the second product maybe it will cause a problem for her and her child also, she wants to use a cream for her acne and she wanted to Take adapalene. Which is contraindicated for her case. I explained to her why and prescribed her a safe medical facial wash for acne.	Sometimes they don’t listen to me or listens to their doctor and sometimes the pregnant woman wants to take every medication on the shelf because they think if they take more medications and more supplements, she and her baby will be in a good health.		Specialized training or courses that are easy to study. Not like 1000 pages book	Each company must do a specialized training for pregnancy and lactation medication
I didn’t get enough training in that.	I have a simple background regarding medications that are not allowed during pregnancy.	Consult a doctor first	Only a little is allowed during pregnancy.	Do not use any medicine that contains vitamin A. Do not use cough syrup that contains herbal products except ivy.	Fear of its side effects on the child or mother	yes	Consult a specialist doctor during pregnancy and receive medications through him	Providing a file specialized in diseases and safe medications at each stage of pregnancy
brief practice _ including medications- supplements- cosmetics	Critical role mainly in OTC medications	Risk/ benefit based	Risk on mom/ baby, what would be worse: the medication side effect or the medical condition risks	- chosen to topical ttt over oral if available example: use nasal spray instead of oral decongestant especially with hypertension imbalanced patients.	Limited products for pregnant segment – limited pregnancy studies on many medications.	Medications collected from the pharmacy/healthcare center by other family members _ no private area/ council area _ limited access for prescriber contact.	Programming System double check for medication issues	Continuous maternity/ pregnancy courses education
Pregnant women should not be given OTC medications. During the first months, no medications should be given to them except by prescription.	Pretty good	Drug entry into the fetus Risks that lead to miscarriage	The benefit outweighs the risks. The treatment must be taken while preserving the safety of the fetus.	A woman came to me wanting to take naproxen to relieve toothache and she was in the first months of pregnancy. I advised her to take paracetamol, which is safe for pregnant women, but she refused. A woman wanted to take domperidone to stop feeling nauseous, so I advised her to take feminor and she responded to the advice.	Lack of response by women and taking the treatment despite the risks_x000D_ _x000D_ or stopping the medication and not treating it_x000D_ _x000D_ Excessive obsessive thoughts_x000D_ _x000D_	Some women hide their pregnancy even from the pharmacist, even after asking.	Providing pharmacists with an annually updated list of medications that are contraindicated in pregnancy and developing simple tests to ensure understanding.	Continuing Education
Summer training at the Women’s and Maternity Hospital	An important role in preventing side effects and serious effects on the mother and fetus	Not prescribing medications that harm the fetus and the mother – knowing the mother’s other diseases	Depending on the pregnant woman’s health condition	She was using Acretin Quick during the first month of pregnancy and was advised to stop taking it to avoid exposing the fetus to risks.	Unconvinced about the dangers of topical medications	No	Distributed leaflets – reviewing the pregnant woman’s file and asking her about her general health and pregnancy status	Providing a drug consultation clinic

A thematic breakdown of pharmacists’ responses regarding their training, perceived roles, responsibilities, approaches to risk–benefit assessment, counseling practices, challenges, barriers, and recommendations. Illustrative statements highlight key insights and emphasize areas such as training gaps, patient counseling, reliance on evidence-based resources, and the need for structured support systems.

ACOG = American College of Obstetricians and Gynecologists, DIC = Drug Information Center, FDA = Food and Drug Administration (mentioned in context of pregnancy categories), LactMed = Drugs and Lactation Database, Micromedex = IBM Micromedex Clinical Knowledge Database, OTC = Over the Counter, PLLR = Pregnancy and Lactation Labeling Rule, UpToDate = Clinical decision support resource.

### 3.3. Pharmacists’ role and responsibilities

Pharmacists acknowledged their critical role in ensuring the safe use of medications during pregnancy. Their responsibilities included assessing the risks and benefits of medications based on clinical guidelines and patient-specific factors, providing accurate counseling on medication safety, appropriate dosages, and potential side effects, and collaborating with physicians and other healthcare providers to optimize treatment decisions. Additionally, pharmacists played a key role in educating pregnant women on how to safely use medications across different trimesters, as shown in the thematic breakdown of pharmacists’ perceptions and responses regarding prenatal medication management in Table 2.

*“Our role is to ensure the quality and safety of medications for pregnant women.”*

*“We should consider the pregnancy period and provide pharmaceutical advice tailored to each trimester.”*

### 3.4. Risk assessment and decision-making

Pharmacists relied on various sources to assess the risks and benefits of medications, including published research, clinical guidelines such as the Food and Drug Administration Pregnancy Categories and World Health Organization recommendations, and patient-specific factors such as medical history and pregnancy trimester. They frequently referred to teratogenic risk classifications to determine medication safety and to provide evidence-based recommendations.

*“The diagnosis should be clear before assessing the safety of medications.”*

*“I always consider the pregnancy category of the drug before advising on its use.”*

Despite these efforts, some pharmacists expressed uncertainty when advising on less commonly used medications, citing limited access to updated clinical resources and training.

### 3.5. Challenges and barriers in counselling

While pharmacists played an important role in prenatal medication counseling, they also encountered several challenges and barriers in their practice. The most significant barriers included a lack of standardized guidelines, patient hesitancy due to fear of harming the baby, and misinformation from online sources that often led to distrust in pharmacists’ recommendations.

*“Patients often fear harming their baby, making counselling difficult.”*

*“Lack of familiarity with some medications limits my ability to provide confident recommendations.”*

Additionally, pharmacists noted communication challenges arising from time limitations in busy healthcare settings and cultural beliefs influencing medication adherence. These higher-order thematic categories are summarized in Table 3.

**Table 3:** Thematic categories of pharmacists’ responses.

Higher-order category	Summary of key themes	Representative quotes
Training & knowledge	Most pharmacists reported limited or no formal training on prenatal medication management. Self-education and university studies were the primary sources of knowledge.	“There is no self-training, only what I learned during my university studies.”
Pharmacists’ role & responsibilities	Pharmacists perceive their role as crucial in ensuring medication safety during pregnancy, assessing risks, and counseling patients on safe medication use.	“Our role is to ensure the quality and safety of medications for pregnant women.”
Risk assessment & decision-making	Pharmacists assess medication risks using published research, clinical guidelines, and patient-specific factors. Common recommendations include folic acid use and avoiding certain drug interactions.	“The diagnosis should be clear before assessing the safety of medications.”
Challenges & barriers	Challenges include lack of standardized guidelines, patient hesitancy, misinformation, and emotional sensitivity during counseling. Communication barriers were noted due to hormonal changes and anxiety in pregnant women.	“Patients often fear harming their baby, making counseling difficult.”
Resources & recommendations	Pharmacists emphasized the need for structured training programs, better clinical resources, and access to updated drug safety guidelines to improve prenatal medication management.	“We need more reliable resources and continuous professional training on prenatal medication safety.”

Summary of higher-order themes with representative quotes on training, roles, risk assessment, challenges, and resource needs.

### 3.6. Resources and training needs

Pharmacists emphasized the need for training programs, access to up-to-date drug safety databases, and continuing education programs focused on prenatal medication safety. Many suggested more institutional support through hospital-based training programs, standardized guidelines, and interdisciplinary collaborations with obstetricians and healthcare providers, as shown Table 3 which summarizes these higher-order in thematic categories.

*“We need more reliable resources and continuous professional training on prenatal medication safety.”*

*“Hospitals should provide education programs focused on medication use during pregnancy.”*

## 4. Discussion

This study explored pharmacists’ perceptions, roles, and challenges in managing prenatal medication exposure. The findings highlight significant knowledge gaps, barriers in patient counseling, and the need for standardized guidelines. Pharmacists acknowledged their responsibility in medication safety during pregnancy; however, faced several challenges, including insufficient training, limited access to updated clinical resources, and patient uncertainty influenced by misinformation. These findings emphasize the urgent need for improved pharmacist education, interdisciplinary collaboration, and policy development to enhance pharmacists’ ability to support maternal healthcare effectively. Given the small sample size and the Saudi Arabian healthcare context, these findings should be interpreted as context-specific rather than broadly generalizable.

The findings of this study are consistent with present literature, the important role of pharmacists in prenatal medication counseling while also showing the insufficient training and limited access to resources that reduce their effectiveness in this area. Several studies have reported that many pharmacists lack formal training on medication use in pregnancy, leading them to rely heavily on self-education and online resources.^[21,22]^ In fact, pharmacists often refer to non-specialized clinical databases for risk assessment due to insufficient training in pregnancy-related pharmacotherapy.^[23]^ Consequently, pharmacists depend on their personal experiences and available clinical guidelines in the absence of structured educational programs when advising pregnant women. Another important finding of this study is the challenges pharmacists encounter when counseling pregnant women. Many pharmacists noted that patients often fear harming their baby, leading to reluctance in taking prescribed medications, even when necessary. Several studies have shown that pregnant women have strong preference for avoiding medications, even for chronic conditions, due to concerns about potential fetal adverse effects.^[24–26]^ The presence of misinformation from online sources further increases this hesitancy, as pregnant women are frequently exposed to conflicting and unverified medical advice on the internet.^[27–29]^ These concerns emphasize the importance of pharmacist education programs that equip them with clear, evidence-based information, enabling them to effectively address medication related fears and misinformation when counseling pregnant women.

Additionally, pharmacists need reliable and consistent source of information regarding in pregnancy-related medication management remains a major limitation in practice.^[30,31]^ Several studies have indicated that pharmacists face challenges in making informed decisions due to inconsistencies in drug safety classifications and a lack of pregnancy-specific risk assessment tools.^[23,32,33]^ For instance, some generally used drug classification systems, such as the Food and Drug Administration Pregnancy Categories, have undergone changes, yet many pharmacists still rely on outdated frameworks due to limited access to recent updates.^[34,35]^ This inconsistency highlights the need for universally accepted, guidelines that provide clear and evidence-based recommendations on medication use in pregnancy. The findings of this study emphasize the need for structural improvements in pharmacist education, training, and interdisciplinary collaboration. Addressing the identified training gaps requires integrating pregnancy-related pharmacotherapy into both undergraduate courses and continuing professional development. These programs should emphasize risk assessment, evidence-based decision-making, and communication strategies, and be regularly updated to reflect current clinical guidelines.

From a policy perspective, national and international healthcare authorities should prioritize developing standardized guidelines on prenatal medication safety. The establishment of clear, evidence-based recommendations on medication use during pregnancy would ensure consistency in pharmacists’ practice across different healthcare settings. Additionally, the creation of pharmacist-specific decision support tools, such as mobile applications or online databases, could facilitate quick and reliable access to pregnancy-related medication safety information. Furthermore, this study highlights the need for more interdisciplinary collaboration between pharmacists, obstetricians, and other healthcare providers. While pharmacists play an essential role in risk assessment and patient counseling, their contributions are often overlooked in prenatal care settings. Pharmacists should be integrated into maternal health teams to enhance medication safety, improve treatment adherence, and optimize maternal and fetal health outcomes.

One of the strengths of this study is its qualitative approach, which enabled an in-depth exploration of pharmacists’ experiences, challenges, and recommendations related to prenatal medication management. However, several limitations should be acknowledged. The sample size was limited to 18 pharmacists and data were collected via a self-administered online survey, which may have reduced the depth of responses compared to in-person interviews or focus groups. Despite the small sample, thematic saturation was achieved, and this size is consistent with qualitative research standards. Nevertheless, a larger and more diverse sample from different regions and healthcare settings, could provide a more comprehensive understanding of the challenges pharmacists faces. The reliance on self-reported data introduces the potential for recall bias and social desirability bias, as participants may have reported what they perceived as appropriate rather than describing their routine practices. Lastly, because the study was conducted within a specific geographic context, the findings may not be generalizable to other countries with different healthcare systems and pharmacist roles. Future research should include multi-center studies and incorporate interviews or focus groups to enrich understanding of pharmacists’ roles in prenatal care.

## 5. Conclusion

Pharmacists play an important role in supporting medication safety during pregnancy; however, they face many challenges, including limited training, absence of standardized guidelines, and patient hesitancy regarding medication use. This exploratory study offers context-specific insights into these issues, based on the experiences of pharmacists practicing in Saudi Arabia. While the findings suggest a need for structured training, improved access to evidence-based resources, and enhanced interdisciplinary collaboration, these implications should be interpreted cautiously. Future research involving broader and more diverse samples is needed to validate these observations and inform the development of targeted educational and policy initiatives aimed at strengthening pharmacists’ contributions to maternal healthcare. These observations reflect the perspectives of a small, context-specific sample and should be confirmed in larger, more diverse studies.

## Author contributions

**Conceptualization:** Fahad S. Alshehri, Nasser M. Alorfi, Ahmed M. Ashour, Moosa H. Atwadi, Abdulaziz Mohammad Alghamdi, Mohammed M. Aldurdunji, Shaker T. Alsharif.

**Data curation:** Fahad S. Alshehri, Nasser M. Alorfi, Ahmed M. Ashour, Saad M. Wali, Moosa H. Atwadi, Abdulaziz Mohammad Alghamdi, Mohammed M. Aldurdunji, Shaker T. Alsharif.

**Formal analysis:** Fahad S. Alshehri, Shaker T. Alsharif.

**Funding acquisition:** Fahad S Alshehri.

**Investigation:** Fahad S. Alshehri, Shaker T. Alsharif.

**Methodology:** Fahad S. Alshehri, Nasser M. Alorfi, Mohammed M. Aldurdunji.

**Project administration:** Fahad S Alshehri.

**Resources:** Fahad S. Alshehri, Saad M. Wali.

**Software:** Fahad S. Alshehri.

**Supervision:** Fahad S. Alshehri.

**Validation:** Fahad S. Alshehri.

**Visualization:** Fahad S. Alshehri.

**Writing – original draft:** Fahad S. Alshehri, Nasser M. Alorfi, Shaker T. Alsharif.

**Writing – review & editing:** Fahad S. Alshehri, Nasser M. Alorfi, Ahmed M. Ashour, Saad M. Wali, Moosa H. Atwadi, Abdulaziz Mohammad Alghamdi, Mohammed M. Aldurdunji, Shaker T. Alsharif.

## Supplementary Material




