# Supplementary material for: Pharmacists’ roles and perceptions in managing prenatal exposure to medications: A qualitative study
Source: Medicine (Baltimore). 2025 Oct 31;104(44):e45618. doi: 10.1097/MD.0000000000045618 (PMC12582782; doi:10.1097/MD.0000000000045618)
Supplement: Supplementary file 1 [file medi-104-e45618-s001.pdf]

Pharmacists' Roles and Perceptions in Managing Prenatal Exposure to Medications: A Qualitative Study

Demographic Information:

1. What is your role and how many years of experience do you have in pharmacy practice?

Knowledge and Training:

2. Describe the training you've received regarding medication management during pregnancy.

Perceptions and Roles:

3. How do you perceive your role in managing prenatal exposure to medications?
4. What do you consider the key responsibilities when advising pregnant women about medication?

Practice and Advising:

5. How do you assess the risks and benefits of medications prescribed to pregnant women?
6. Can you provide an example of how you advised a pregnant woman about her medication use?

Challenges and Barriers:

7. What challenges do you commonly encounter in advising pregnant women on medication?
8. Are there barriers that hinder effective communication about medication safety during pregnancy?

Improvements and Recommendations:

9. What tools or resources would improve your management of prenatal medication exposure?
10. What changes would enhance pharmacists' abilities to advise pregnant women effectively?
